# Association of Surgical Debridement Timings With Infection and Non-union Rates in Open Fractures of Lower Limb Long Bones

**DOI:** 10.7759/cureus.77392

**Published:** 2025-01-13

**Authors:** Amandeep S Bakshi, Jagdeep S Rehncy, Mukul Sharma, Jaspreet Singh, Abhishek Nanda, Harry Mehta

**Affiliations:** 1 Orthopaedics, Government Medical College and Hospital, Patiala, Patiala, IND

**Keywords:** gustilo anderson classification, nonunion, open fractures, orthopedic infections, timing of debridement

## Abstract

Background: Open fractures are one of the orthopaedic conditions that require urgent surgical intervention. Managing these fractures remains challenging for orthopaedic surgeons due to the need to transfer polytrauma patients to hospitals with advanced capabilities. Further delays may occur as resuscitative life-saving measures take precedence. Open fractures are frequently complicated by infections, non-unions, and, in rare cases, amputation. Currently, management of compound fractures of long bones of the lower limb requires early surgical debridement followed by limb salvage procedures or amputation (if required), depending on the type, location, and extent of the injury. Early and aggressive debridement of open fractures has always been the rule.

Objectives: To study the role of timing of surgical debridement of open fractures of the lower limb and its effect on infection and non-union rates and to analyze the impact of increased severity of open fractures on union and infections.

Materials and methods: The study was conducted prospectively in the orthopaedic department of a tertiary care hospital of Patiala with a population of 223 patients who presented to the orthopaedic emergency department with open lower limb fractures. Patients were divided into two groups based on the timing of surgical debridement: Groups A and B. Group A consisted of the patients who were operated on within 24 hours (n=110) and Group B consisted of patients whose surgical debridement was conducted 24 hours after injury (n=113). Infection rates and non-union rates were obtained based on the above data. All the results were summarized in Microsoft Excel (Microsoft Corp., Redmond, WA) and were analyzed with SPSS software 22 (IBM Corp., Armonk, NY) using the ANOVA test, chi-square test, and paired t-test. The Gustilo-Anderson classification (GAC) was used to classify the grades of open fractures. A p-value <0.05 indicated a statistically significant difference.

Results: The mean age in Group A was 39.53±13.25 years (range 18-80) and the mean age in Group B was 42.45±12.64 years (range 18-76) (p=0.0936; not significant) In Group A, infection was present in 30 patients (27.27%) and in Group B, infection was present in 32 patients (28.32%) (p=0.9802; non-significant). Non-union was present in eight patients (7.27%) and 13 patients (11.50%) in Groups A and B, respectively (p=0.2793; non-significant). In Group A, the infection rate was 0% for GAC Grade 1, 10% for Grade 2, 35.89% for Grade 3A, and 66.67% for Grade 3B (p-value < 0.00001; statistically significant). In Group B, the infection rate was 2.86% for GAC Grade 1, 13.79% for Grade 2, 57.69% for Grade 3A, and 52.17% for Grade 3B (p-value < 0.00001; statistically significant). In Group A, the non-union rate was 0% for GAC Grade 1, 0% for Grade 2, 7.69% for Grade 3A, and 23.81% for Grade 3B (p-value < 0.00001; statistically significant). In Group B, the non-union rate was 0% for GAC Grade 1, 6.89% for Grade 2, 19.23% for Grade 3A, and 26.09% for Grade 3B (p-value < 0.00001; statistically significant).

Conclusion: The timing of surgical debridement in open fractures of the lower limb does not have a significant role in their management and these fractures can safely be debrided up to several hours after injury. GAC grading of open fractures has a significant association with infection and non-union rate, which increased significantly with increasing grades of open fractures.

## Introduction

A bone fracture is a breach in the structural continuity of the bone cortex, with a degree of injury to the surrounding soft tissues [1]. Despite being a widespread public health concern, there has not been a thorough examination of the frequency and impact of bone fractures [2]. In recent years, there has been a rise in the occurrence of long bone fractures, mainly due to accidents on the road. Various factors determine the likelihood of infection after an open fracture. The severity of the wound is the most notable among them. Increased soft-tissue damage combined with compromised blood flow creates conditions that are more favorable for bacterial growth and infection to occur. The level and nature of contamination dictate the amount of bacteria present and the specific species that may cause infection. The majority of infections seem to stem from exposure within healthcare facilities [3]. Patient habits and characteristics such as smoking, weakened immune system, and diabetes have been linked to higher vulnerability to infections [4,5]. When a fracture occurs, it is categorized as either an open or closed fracture. An open fracture (also called compound fracture) occurs when the bone protrudes through the skin or a deep wound exposes the bone. Closed fracture (also called simple fracture) is one in which the bone is fractured, yet the skin remains unharmed [6].

Even with multiple classification systems in place, there is no single system that effectively categorizes open fractures according to their outcomes and accurately predicts their prognosis [7]. The Gustilo-Anderson classification (GAC), introduced in 1976, has been the primary method for categorizing open fractures [8]. It considers the severity of the wound, wound size, periosteal stripping, wound contamination, and vascularity status, but has limitations in consistency and sensitivity [9].

Gustilo et al. outlined three main categories: Types I-III, based on wound contamination, surrounding soft-tissue injury, and skin wound size. Type I fractures are defined as clean wounds with minimal contamination, with a skin wound < 1 cm diameter and a simple fracture pattern, e.g., simple transverse fractures or short oblique fractures. Type II fractures have wounds > 1 cm but < 10 cm in diameter with moderate soft-tissue damage with moderate contamination and adequate bone coverage, e.g., simple transverse fractures or short oblique fractures. Type III fractures involve those that are open with a significant tear (> 10 cm), injury or absence of soft tissue, or an open fracture with a section that is fractured. This category also covers open fractures from farm accidents, fractures needing vascular surgery, or fractures left open for eight hours before receiving treatment. High-energy fractures demonstrate a significant impact on surrounding tissue [10].

Furthermore, Gustilo et al. proposed Types IIIA, IIIB, and IIIC based on the amount of wound coverage required and signs of vascular impairment: (a) Type IIIA fractures comprise fractures with adequate soft tissue coverage with extensive wound contamination regardless of the size of the wound; (b) Type IIIB fractures involve severe damage to soft tissues with stripping of periosteum and exposure of bone (usually associated with extensive contamination); (c) Type IIIC consists of open fractures that are associated with vascular injury requiring urgent repair [10].

Because an open fracture is defined as an injury in which either the fractured bone or hematoma (or both) are visible to the outside environment, it presents an immediate danger of infection and necrosis. Therefore, immediate treatment is essential [11]. The primary goals of the treatment focus on restoring limb function and avoiding the negative effects of infection and non-union [7]. It is crucial to irrigate the open fracture wound with sterile normal saline to eliminate bacteria and promote wound healing, along with using systemic antibiotics and an antibiotic bead pouch for Grade III B and III C fractures [12]. There is a lack of literature to provide insight regarding the infection rates in open fractures operated on after 24 hours of injury. Therefore, we conducted this study to prospectively examine the association between the time from injury to debridement of open fractures and associated complications. Specifically, we examined the effect of both early and delayed debridement (>24 hours) on complications such as infections and non-unions.

## Materials and methods

The present study was a prospective study conducted in the Department of Orthopedics, Rajindra Hospital, Government Medical College, Patiala. A total of 223 patients who presented to the emergency department were included in the study sample. Informed written consent was taken from all the patients/ patient’s attendants. The enrollment was based on the following inclusion criteria: adult skeletally mature patients (seen on radiographs) of age > 17 years, open fractures of long bones of the lower limb, and patient/relatives able to provide consent. The exclusion criteria were isolated fractures of the bones of the foot, pelvis, patella, and spinal trauma, surgery/debridement done elsewhere, and patients/ relatives not giving consent / unwilling to participate in the study.

Following enrollment using stringent selection criteria, patients underwent surgical debridement either within the first 24 hours post-injury (Group A, 110 patients) or after 24 hours post-injury (Group B, 113 patients). Deep infections are defined as wounds requiring unplanned debridement and/or sustained antibiotic therapy following definitive closure [13-14]. Wound healing was evaluated by examination of the wound to determine if it was healed or not. An ideally healed wound results in a return to normal anatomical structure, appearance, and function that includes a fully differentiated dermis and epidermis with barrier function intact. An acceptably healed wound is characterized by epithelization capable of sustaining functional and structural integrity. A minimally healed wound is characterized by epithelial coverage restoration that does not establish a sustained functional result and recurrence may occur. A time limit of one month following injury was considered to assess the healing of the wound. A positive pus culture from the wound also confirmed the presence of infection. Assessment of blood CRP marker one month post-operation was also considered. Studies have shown that CRP levels help in the diagnosis of infections when they are not clinically evident [15].

All patients underwent clinical and radiological evaluation at nine months to determine union, the US FDA-defined maximum time period for union in 1986. As per the FDA, a bone fracture that hasn't fully healed after nine months post-injury and hasn't shown signs of healing on X-rays for three consecutive months should be classified as a non-union. X-ray imaging was used to identify a non-union based on specific criteria: no bony trabeculae crossing the fracture site, sclerotic fracture margins, continued fracture lines, less than 75% cortical healing, and no advancement on successive X-rays towards union [16]. Other than this, clinical signs such as inability to bear full weight, persistent pain, abnormal mobility at the fracture site, and pain when palpated or on bearing weight were also considered for diagnosis.

The results of observations of individual patients were pooled into two groups and analyzed. Statistical analysis was performed using SPSS software v. 20.0 (IBM Corp., Armonk, NY). All the analyses were performed on an intention-to-treat basis. A chi-square test was conducted for the analysis of gender distribution, and comparison of infections and non-union rates among both the groups, a paired t-test was used for age distribution, and an ANOVA test was used for the analysis of the relationship of GAC grade with infections and non-union rates. The p-value was calculated as the difference between the two groups which would have arisen by chance. If it was less than 0.05, it was considered significant and if it was more than 0.05, it was considered non-significant.

## Results

In Group A, 30 patients (27.27%) had Grade 1 fractures, 20 patients (18.18%) had Grade 2, 39 patients (35.45%) had Grade 3A, and 21 patients (19.09%) had Grade 3B. In Group B, 35 patients (31.82%) had Grade 1 fractures, 29 patients (26.36%) had Grade 2, 26 patients (23.64%) had Grade 3A and 23 patients (20.35%) had Grade 3B.

The mean age in Group A was 39.53±13.25 years (range 18-80) and the mean age in Group B was 42.45±12.64 years (range 18-76) (p=0.0936; not significant) (Table 1).

**Table 1:** Distribution of patients according to age Paired t-test is used

Age	Group A					Group B			
	Frequency		Percent			Frequency			Percent
≤20	5		4.54%			5			4.42%
21-40	57		51.82%			46			40.71%
41-60	41		37.27%			54			47.79%
61-80	7		6.36%			8			7.08%
Total	110		100%			113			100%
Range	18-80					18-76			
Mean (±SD) age	39.53±13.25					42.45±12.64			
p-value	0.0936 (not significant)								

Table 2 shows that in Group A, there were 15 (13.64%) females and 95 (86.36%) males. In the Group B, there were 11 (9.73%) females and 102 (90.26%) males (p=0.3640; not significant).

**Table 2:** Distribution of patients according to gender Chi-square test is used.

Gender	Group A		Group B	
	Frequency	Percent	Frequency	Percent
Male	95	86.36%	102	90.26%
Female	15	13.64%	11	9.73%
Total	110	100%	113	100%
X2	0.0304			
p-value	0.3640 (not significant)			

Table 3 shows the comparisons of infection in both groups; in Group A, infection was present in 30 patients (27.27%), and in Group B, infection was present in 32 patients (28.32%) (p=0.9802; non-significant).

**Table 3:** Comparison of Infection in Group A and Group B Chi-square test is used.

Presence of infection	Group A		Group B	
	Frequency	Percent	Frequency	Percent
Present	30	27.27%	32	28.32%
Absent	80	72.73%	81	71.68%
Total	110	100%	113	100%
X^2^	0.0304			
p-value	0.9802 (not significant)			

Table 4 shows that non-union was present in eight patients (7.27%) and 13 patients (11.50%) in Groups A and B, respectively (p=0.2793; non-significant).

**Table 4:** Comparison of non-union in Group A and Group B Chi-square test is used.

Presence of non-union	Group A		Group B	
	Frequency	Percent	Frequency	Percent
Present	8	7.27%	13	11.50%
Absent	102	93.64%	100	88.49%
Total	110	100%	113	100%
X^2^	1.1701			
p-value	0.2793 (not significant)			

Table 5 shows the relationship between GAC grade with infections. In Group A, two patients (10%) with Grade 2 had infections, 14 patients (35.89%) with Grade 3A had infections, and 14 patients (66.67%) with Grade 3B had infections. The relationship was statistically significant (p < 0.00001). In Group B, one patient (2.86%) of Grade 1 had infections, four patients (13.79%) with Grade 2 had infections, 15 patients (57.69%) with Grade 3A had infections, and 12 patients (52.17%) with Grade 3B had infections. The relationship was statistically significant (p < 0.00001).

**Table 5:** Relationship of Gustilo-Anderson grade with infections ANOVA test is used. GAC: Gustilo-Anderson classification; ANOVA: analysis of variance

GAC grade	Presence of infection			
	Group A		Group B	
	Frequency (total)	Percent	Frequency (total)	Percent
Grade 1	0 (30)	0%	1 (35)	2.86%
Grade 2	2 (20)	10%	4 (29)	13.79%
Grade 3A	14 (39)	35.89%	15 (26)	57.69%
Grade 3B	14 (21)	66.67%	12 (23)	52.17%
p-value	< 0.00001 (significant)		< 0.00001 (significant)	

Table 6 shows the relationship between GAC grade with non-union. In Group A, three patients (7.69%) with Grade 3A had non-union, and five patients (23.81%) with Grade 3B had non-union. The relationship was statistically significant (p < 0.00001). In Group B, two patients (6.89%) with Grade 2 had non-union, five patients (19.23%) with Grade 3A had non-union, and six patients (26.09%) with Grade 3B had non-union. The relationship was statistically significant (p < 0.00001).

**Table 6:** Relationship of GAC grade with non-union ANOVA test is used. GAC: Gustilo-Anderson classification; ANOVA: analysis of variance

GAC grade	Presence of non-union			
	Group A		Group B	
	Frequency (total)	Percent	Frequency (total)	Percent
Grade 1	0 (30)	0%	0 (35)	0%
Grade 2	0 (20)	0%	2 (29)	6.89%
Grade 3A	3 (39)	7.69%	5 (26)	19.23%
Grade 3B	5 (21)	23.81%	6 (23)	26.09%
p-value	< 0.00001 (significant)		< 0.00001 (significant)	

## Discussion

Bone fractures represent a significant global public health concern; however, comprehensive studies assessing their incidence and burden are lacking. Open fractures, characterized by the bone protruding through the skin or a deep wound exposing the bone, require effective classification systems to inform prognosis and treatment outcomes. Open fractures of lower limbs are the most prevalent long-bone injuries, and their management continues to be controversial. Treatment objectives focus on maximizing limb function while minimizing the risks of complications such as the development of infections and non-union [7]. In our study, the majority of the patients involved in compound fractures were young adults (age group: 21-40 years). Out of 223 patients, the majority were males (88.3%) possibly signifying that the local male population may be more involved in outdoor activities exposing themselves to a higher risk of traumatic injuries.

Gustilo-Anderson grade

In Group A, 30 patients (27.27%) had Grade 1, 20 patients (18.18%) had Grade 2, 39 patients (35.45%) had Grade 3A, and 21 patients (19.09%) had Grade 3B. In Group B, 35 patients (31.82%) had Grade 1, 29 patients (26.36%) had Grade 2, 26 patients (23.64%) had Grade 3A and 23 patients (20.35%) had Grade 3B. Singh et al. (2023) documented that the highest number of patients had open Grade 2 fractures (39%), followed by open Grade 3 fractures (33%). Additionally, 15.38% of patients experienced Grade 3A fractures, 13.84% had Grade 3B fractures, 3.84% presented with Grade 3C fractures, and 27% had Grade I fractures [17]. Joseph et al. (2020) reported that 65% of the fractures were Grade 3B, 31% of the fractures were Grade 3A, and 4% of the fractures were 3C [18]. Increasing grades of injuries shown in large numbers suggest that high-velocity/high-impact trauma cases are on the rise owing to advancements in modern technology and people’s fast-paced lifestyles.

Infections

In our study, in Group A, infection was present in 30 patients (27.27%) and in Group B, infection was present in 32 patients (28.32%) (p 0.9802; non-significant). Thus our study concludes that the timing of debridement does not have a significant role in the development of infections even if surgery is delayed beyond 24 hours. Singh et al. (2023) indicated that the infection rates for fractures that were debrided within six hours were 18.75%, while the rate for the 6-12 hours group was 18.50%, and for the 12-24 hours group, it was 14.28%. If surgical debridement was performed after 24 hours, the infection rate rose to 38.8%. However, statistical analysis revealed that the timing of debridement was not a significant factor [18]. Similarly, Solomon et al. (2021) found that among 44 patients in Group A (patients who underwent early debridement), 21 developed infections, while in Group B (late debridement group), which included 45 patients, 19 experienced infections over the course of a year. Statistical analysis indicated no significant association between the timing of debridement and the infection rate, with a p-value of 0.6 [19]. Hendrickson et al. (2018) also found no significant differences in the incidence of infective complications between patients operated in less than 12 hours compared to those debrided after 12 hours, with rates of 4.5% and 5.6%, respectively (p = 1.00) [20].

Infection represents a significant concern for orthopaedic surgeons managing open fractures, as it is a common complication associated with considerable tissue damage and contamination. The presence of infections can lead to severe complications, including limb dysfunction, multiple organ failure, and even mortality. Consequently, numerous strategies, primarily focused on emergency interventions, have been proposed to decrease the risk of infective complications in patients with open fractures. Among these strategies, early debridement within six hours post-injury is widely regarded as the most effective method for controlling infection; however, achieving this timeline can be challenging in practice due to the several conditions often present in such cases [21]. This six-hour rule for debridement appears to have historic significance by virtue of advancements made in antibiotic development and their impact on preventing infections [17].

In our study, delayed debridement resulted from various factors, including delayed patient presentation. Since our study centre functions as a referral centre, patients were first taken to local primary care centres for initial first aid. Additionally, polytrauma patients requiring emergency resuscitation or neurosurgical clearance often experienced further delays in the debridement of open fractures, as these life-saving procedures took precedence.

Non-union

In our present study, non-union was present in eight patients (7.27%) and 13 patients (11.50%) in Groups A and B, respectively (p=0.2793; non-significant). Thus our study concludes that timing of debridement does not have a significant role in the development of non-union in open fractures. A study by Singh et al. (2012) showed that five of 38 patients in the early debridement group had non-union, and one of 29 patients in the late debridement group experienced non-union [22]. 

Limitations

The limitations of this study were, despite the relatively long follow-up periods and completeness of data, we acknowledge that we may not have analyzed other variables that may have influenced the rate of infection, for example, the timing of definitive wound closure; the type of antibiotics given; the method of fixation of the fracture, the technique of irrigation of wound, confounding factors related to patient (smoking, diabetes status), fracture, and choice of fixation methods. For this, we may require a larger sample size. Therefore, we recommend that the results of our study be confirmed with multicentric large-sample studies.

## Conclusions

Our study shows that the timing of surgical debridement in open fractures of lower limbs does not have a significant role in their management and these fractures can safely be debrided up to several hours after injury. Gustilo and Anderson's grading of open fractures significantly affects infection and nonunion rates. Infection and nonunion rates increased significantly with increasing grades of open fractures. Further, we conclude that debridement can be performed once the patient is adequately resuscitated and stable for surgery as these surgeries can be done safely even several hours after the injury. The process of wound coverage and definitive fixation begins with the initial debridement, focusing on the stability of bony architecture and prevention of infection, also taking into account the comorbidities of the patient and overall nutritional and health status. The combined experience of the orthopedic and plastic surgeon in assessing the bony injury and soft tissue injury will improve patient care and favour earlier reconstruction whenever appropriate.
